# Influence of different finishing and polishing protocols of composite CAD CAM blocks on surface roughness and biological response of gingival mesenchymal stem cells

**DOI:** 10.1007/s10266-025-01190-6

**Published:** 2025-09-13

**Authors:** Mohamed F. Haridy, Mohamed Shamel, Raghda A. Khalil, Ahmed Refaat Mohamed, Hoda Fouda, Hend S. Ahmed

**Affiliations:** 1 Department of Conservative Dentistry, Faculty of Dentistry, The British University in Egypt, Cairo, Egypt; 2 Department of Conservative Dentistry, Faculty of Dentistry, Cairo University, Cairo, Egypt; 3 Department of Oral Biology, Faculty of Dentistry, The British University in Egypt, Cairo, Egypt; 4 Dental Science Research Group, Health Research Centre of Excellence, The British University in Egypt, Cairo, Egypt

**Keywords:** CAD/CAM, Composite finishing and polishing, Roughness, Biocompatibility, Mesenchymal stem cells

## Abstract

The surface quality of composite CAD/CAM restorations is vital in aesthetics, longevity, and the biological response of surrounding gingival tissues. Yet, little is known about how different finishing and polishing techniques influence cellular behavior at the tissue interface. This study aimed to evaluate how various finishing and polishing systems affect the surface roughness of composite CAD/CAM blocks, and how these differences influence the attachment, viability, and inflammatory response of gingival mesenchymal stem cells (GMSCs). Seventy-seven composite CAD/CAM specimens were prepared and subjected to one-step, two-step, and multi-step finishing/polishing protocols, with or without polishing paste. Surface roughness was measured using a digital profilometer. GMSCs were isolated from healthy donors, characterized, and cultured on the composite surfaces. Cell viability was assessed by MTT assay, adhesion was evaluated using scanning electron microscopy (SEM), and inflammation-related gene expression (IL-1β and TGF-β) was analyzed via q-PCR. Polished surfaces significantly reduced roughness and improved biological outcomes. Two-step finishing and polishing with paste resulted in the smoothest surfaces. GMSCs showed greater attachment and viability on polished composites, particularly in the two-step group. Inflammatory gene expression was lowest in polished groups, with IL-1β expression highest in unpolished specimens and TGF-β expression highest in the one-step groups. Finishing and polishing protocols directly impact the surface roughness as well as the biocompatibility of CAD/CAM composite surfaces. Smoother surfaces achieved through proper finishing and polishing enhance cell attachment and viability, and reduce inflammatory responses, highlighting the critical role of finishing in restorative success beyond aesthetics.

**Supplementary Information:**

The online version contains supplementary material available at 10.1007/s10266-025-01190-6.

## Introduction

Computer-aided design and computer-aided manufacturing (CAD/CAM) blocks have been introduced as an easier and faster replacement for direct restorations. This technology reduced the number of defects in the restorations and decreased pores by increasing the material homogeneity. This is achieved by amplification of the volume of filler content and other modifications in the matrix types [1].

Indirect restorations can eliminate some of the clinical difficulties that the operator faces when using direct resin composite. Such challenges include moisture control, technique sensitivity, polymerization shrinkage, residual monomers, and degree of monomer conversion during curing [2]. Besides the introduction of ceramic CAD/CAM blocks, another breakthrough in digital dentistry is the manufacturing of resin-based CAD/CAM blocks.

Recently, resin-based composite CAD/CAM blocks, such as reinforced composite blocks or hybrid ceramics, such as polymer-infiltrated ceramic network, demonstrated better mechanical and comparable aesthetic properties [1]. Incorporation of ceramic fillers in the resin matrix for both direct and indirect restorative materials results in a high modulus of elasticity and enhanced wear resistance in the opposing teeth [3, 4]. The high mechanical properties of CAD CAM composite blocks are owing to their high polymerization degree due to the optimized curing methodology, such as curing under high temperature, using multiple curing light sources, curing in an oxygen-free environment, and high filler loading with better silanisation [5, 6]. This property makes such blocks more favorable than ceramic ones in parafunctional occlusion cases. Moreover, such blocks could be repaired intraorally [4]. They are also characterized by their marginal integrity, short milling time, fewer voids, and high edge strength. These properties made the resin-based restorations a strong rival to ceramic-based restorations [7].

However, degradation remains a drawback in resin-based restorations compared to ceramic restorations, which are always related to the resin matrix. Moreover, the oral environment itself is considered a threat that is sculptured by smoking, daily beverages as tea and coffee, and food [8]. Furthermore, plaque accumulation, gingival, and periodontal inflammation near the unfinished restorations are usually accompanied by discoloration in most restorations. Resin-based materials present a particular challenge in aesthetics and soft tissues, particularly the gingival tissues, attachment, and biological reaction close to the restorations, which is crucial to their success [9].

Gingival health is a critical component of overall oral health. The interface between dental materials and gingival tissues can significantly impact the biological responses of these tissues, including the behavior of gingival stem cells. These cells are essential for maintaining and repairing the periodontal ligament and surrounding mucosal tissues, making their interaction with dental materials a key area of investigation [9, 10].

Different finishing and polishing techniques are employed to remove the easily roughened, stain-prone outer resin layer created by filler exposure in the mouth due to the uncured, resin-rich surface layer [11]. Material selection and technique significantly impact the finishing and polishing procedures in dentistry, with diverse options like strips, rubber cups, diamond instruments, and polishing paste contributing to the outcome. Different shapes and systems of finishing and polishing have been introduced to enhance the efficiency of the procedure [11]. The efficiency and time consumption of a finishing and polishing system depend on several factors, including the type of restoration, the desired surface finish, and the number of steps in the system [12].

One-step finishing systems are designed to save time and improve efficiency by combining multiple steps into one [13]. On the other hand, two-step finishing and polishing systems involve two discs or tools, each with a combination of abrasive grits to finish and polish a restoration. These systems are typically more efficient, but more time-consuming than a one-step system. However, compared to the multistep finishing and polishing systems, they reduce procedure time by minimizing tool changes and cleaning. Multistep finishing and polishing systems typically involve three to four different discs or tools, each with different abrasive sizes [14]. Another factor that may also affect the efficiency of the finishing and polishing step is the application of polishing paste according to each system, depending on the manufacturer’s recommendations [15].

Surface roughness is considered an important parameter regarding the longevity and success of restorations. Skipping the finishing procedure results in a rough surface, causing an increase in the susceptibility of discoloration and bacterial buildup. Research has shown that achieving a smooth and polished finish on restorations minimizes bacterial attachment, enhances the seal at the restoration margins, improves the material’s adaptation to the tooth structure, and enhances color stability [16, 17].

While it is commonly believed that CAD/CAM blocks have relatively smooth surfaces and an inert biological effect, there have not been many studies exploring how different finishing methods might affect the roughness and biological reaction of these resin-based materials in the form of the cellular functions of gingival stem cells at the molecular level. Therefore, this study aimed to evaluate the effect of different finishing and polishing systems on the surface roughness of composite CAD/CAM blocks and the attachment, viability, and inflammatory response of gingival mesenchymal stem cells (GMSCs). The null hypothesis is that there is no difference in the effect of different finishing and polishing systems on the surface roughness of composite CAD/CAM blocks and the biological reaction of the gingival mesenchymal stem cells.

## Materials and methods

All the materials used, and their specifications, composition, and manufacturer are listed in Table 1.

**Table 1:** Materials’ specifications, composition, manufacturer, and lot number

Material	Specifications	Composition	Manufacturer	Lot number
Brilliant Crios	Nano-hybrid composite blocks	Fillers: Barium glass, size < 1.0 µm; Amorphous silica SiO_2_, size < 20 nm	Coltène Whaledent, Switzerland	135587
		Resin matrix: Cross-linked methacrylates (BISGMA, TEGDMA) and inorganic pigments (ferrous oxide or titanium dioxide)		
ENA diamond polishing paste	Diamond polishing paste	One μ diamond polishing paste	Micerium S.P.A, Avegno (GE), Italy	1904046
Opti1step	One-step composite polisher	Silicone tip impregnated with small diamond particles mounted on a metal rod	Kerr Corporation, Orange, USA	5992627
Spiral wheels	Two-step finishing and polishing system for resin composite	Elastomer impregnated with diamond particles	3M, St. Paul, MN, USA	N514708
		Finishing wheels pre-polish (beige)		
		Polishing wheels (pink)		
Composoft	Multi-step polisher for resin composite	Aluminum oxide silicone-bonded polishing system for processing composite	EVE Ernst Vetter GmbH, Keltern, Germany	474378

### Sample size calculation

Sample size calculation was achieved using G*Power 3.1.9.4 software based on a previous study by Aydin et al. [18]. Using 80% power and a 5% significance level, a sample size of 11 CAD/CAM composite blocks per group was estimated to detect an effect size of 1.26, for a total of 77 blocks per seven groups.

### Preparation of CAD CAM composite blocks

The resin-based blocks were cut using a water-cooled diamond impregnated disc at a low-speed micro-slicing machine (Isomet 5000, Buehler, Buehler Ltd, Lake Bluff, Illinois, USA) into 2 mm-thick rectangular blocks. The CAD–CAM block was sectioned into two blocks, resulting in the final specimens’ dimensions (12 × 6 × 2 mm). All specimens were measured with a stainless-steel manual caliper to ensure their dimensions. The specimens were thoroughly checked after cutting for defects such as cracks and chipping using magnifying loupes 5x (Ergovision, China). Finally, they were cleaned in the ultrasonic device bath (Coxo, China) with distilled water for 5 min [19].

### Study design and specimen grouping

The study design was granted ethical approval (24-039) from the Faculty of Dentistry, the British University in Egypt. Seventy-seven specimens were derived from eight composite CAD/CAM blocks. Sixty-six blocks were randomly assigned to three study groups (22 each) according to the finishing and polishing system (either one-step, two-step, or multi-step finishing and polishing systems). Each group was subdivided into two subgroups (11 each) based on whether they applied polishing paste (either polishing with diamond polishing paste or without polishing paste). The remaining 11 blocks were not finished and polished to be used as a control group (C).

The groups were assigned as follows:

C: Control group

Ia: One-step with polishing

Ib: One-step without polishing

IIa: Two-step with polishing

IIb: Two-step without polishing

IIIa: Multi-step with polishing

**Fig. 1:**
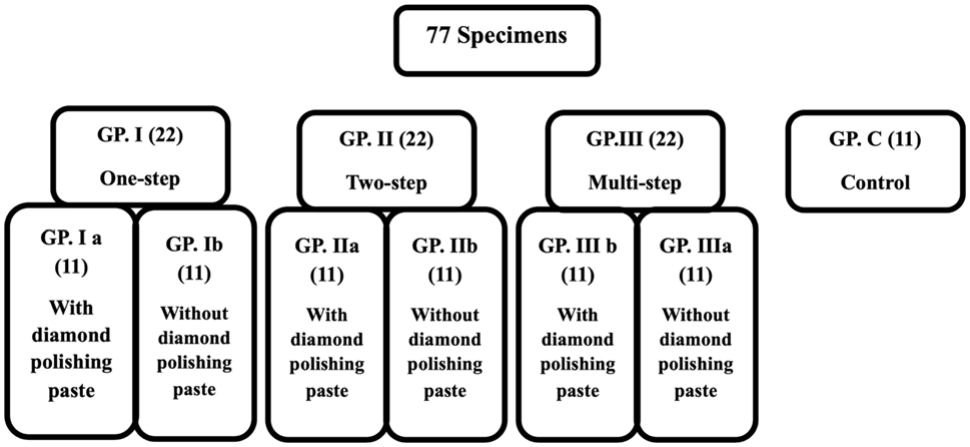
Schematic diagram for grouping of specimens

IIIb: Multi-step without polishing (Fig. 1)

### Finishing and polishing systems

A yellow-coded, tapered finishing stone from Komet (Germany) was used to grind the experimental surface of the composite block for 30 s, simulating the effect of 600–800 grit sandpaper [13]. To obtain a standard finished surface, the blocks were cleaned with distilled water for 10 s in an ultrasonic cleaning device and air dried to standardize the baseline surface in all specimens, representing the pre-finishing step. An electric motor handpiece (NSK, Japan) was used at the manufacturer’s recommended speed, minimizing variability due to equipment differences. Additionally, each polishing tool was employed only once and discarded to avoid the impact of wear and tear on results. All procedures were conducted by a single operator, ensuring consistent technique and pressure application throughout the study, and to provide consistent pressure application, a kitchen scale served as a guide. The operator applied moderate pressure equivalent to 60 g according to manufacturer recommendations [20]. Ten strokes were applied to each surface with precise direction and planar motion for even coverage across all specimens.

Regarding polishing paste, the original syringe was evacuated in a graduated plastic syringe (3 mL) to standardize the amount of polishing paste used with each specimen. All specimens were then cleaned with distilled water for 10 s in an ultrasonic cleaning device.

#### The finishing and polishing procedures were performed as follows

For the control group, no finishing or polishing procedure was performed. For the one-step system, Opti1step (Kerr Corporation, Orange, USA) was used at 17,500 rpm at moderate pressure (60 g) in wet conditions according to the manufacturer’s recommendation. The disc-shaped tool was loaded on an electric handpiece (NSK, Japan) and moved in one direction in planar motion with 10 strokes for each specimen. Moreover, the one-step system group was divided into two subgroups. The first subgroup was without application of polishing paste, while the second one was with polishing paste [ENA, Micerium, S.P.A, Avegno (GE), Italy]. A standard amount of polishing paste (0.1 ml) was applied to the center of the specimens, and then the surface was polished using the previously mentioned parameters. After the procedure, the specimens were rinsed with the triple air–water syringe for 10 s to remove the debris, then air-dried for 5 s. For the two-step system, spiral wheels were used at 17,500 rpm at a moderate pressure (60 g) in wet conditions according to the manufacturer’s recommendation to obtain optimum results. Starting with the pre-polish one (beige color) for the finishing step, followed by the polish spiral wheel (pink) for the polishing step. All steps were done in one direction in a planar motion. Spiral wheels were mounted to the Sof-lex mandril and attached to the electric motor handpiece by the same operator. According to the application of polishing paste, the two-step system group was also subdivided into two subgroups. 0.1 ml of the polishing paste was applied to the center of the specimens, then the surface was polished with the previously mentioned parameters using the pink spiral wheel. After the procedure, the specimens were rinsed with the triple air–water syringe for 10 s to remove the debris, then air-dried for 5 s. For the multi-step finishing and polishing system, Composoft (EVE Ernst Vetter GmbH, Keltern, Germany) was used at 8000 rpm at moderate pressure (60 g) in wet conditions according to the manufacturer’s instructions. The coarse disc-shaped tip (white) was used for smoothening, followed by the medium disc-shaped (blue) for the pre-polishing step, and then the fine disc-shaped (pink) for polishing. All steps were done in one direction in a planar motion. Regarding the polishing paste subgroup, application of polishing paste was done as previously mentioned in the subgroups.

### Surface roughness

Surface roughness (Ra) was measured with a digital surface profile gauge (Elcometer 224/2, Elcometer Instruments, Great Britain), and data were recorded using the computer software of the roughness tester supplier (Elcomaster 2, Elcometer Instruments). Surface roughness was measured for all specimens, including the control group and all finishing and polishing systems with and without polishing paste. For every reading made, the mean roughness value (Ra, μm) was represented by the arithmetic mean between the peaks and valleys registered, after the needle of the profilometer had scanned a stretch of 2 mm in length, with a cut-off of 0.25 mm to maximize the filtering and the undulation on the surface. The surface roughness measurements were performed at the center points of the same samples. Regarding the average of measurements, each surface was read three times, always with the needle scanning the geometric center of the specimen, starting from three different points. Additionally, measurements were performed twice for each sample by rotating it 60 degrees to ensure that the small tip contacted several areas on the surface, and an average of the five measurements was taken. The mean value of the five readings yielded the mean roughness value of each specimen.

### Extract preparation

Composite disc extracts were prepared at a surface area-to-medium volume ratio of 3 mm^2^/mL and diluted 1:2 and 1:4. Discs were incubated in RPMI 1640 medium (Gibco, Grand Island, NY, USA) with 10% FBS in sterilized glass bottles at 37 °C, 5% CO₂ for 24 h. Extracts were then used for cytotoxicity assessment via the MTT assay.

### Gingival mesenchymal stem cells (GMSCs) isolation and culture

The gingival stem cells were isolated from the gingival biopsies of adult healthy patients. The lead investigator provided the patients with a detailed explanation of the study’s goal and allowed them to provide informed consent. The process of isolating stem cells was carried out according to the methodology outlined by Dahak et al. in 2020 [21]. The gingival biopsies were dissected into small fragments and then subjected to enzymatic digestion for 1 h at 37 °C. The digestion was carried out using a mixture containing 3 mg/mL of collagenase and 4 mg/mL of dispase II in a culture medium composed of DMEM/F12 (Dulbecco’s Modified Eagle Medium/F12 Ham medium, Sigma) supplemented with 10% FBS (fetal bovine serum, Gibco BRL, CA, USA) and 1% antibiotic/antimycotic (Gibco). The cells were subsequently placed in a 5% CO_2_ environment at 37 °C, with a humidified atmosphere. The study utilized cells from the fourth passage.

### Characterization and identification of isolated GMSCs

#### Morphology

The morphological alterations and confluency of GMSCs were assessed using an inverted phase microscope (TCM 400, Olympus, Tokyo, Japan). GMSCs were characterized by their fibroblast-like appearance and their capacity to form colonies.

#### Multilineage differentiation potential

GMSCs were cultivated in 12-well plates with a density of 10 × 10^4^ cells per well. The culture medium was supplemented with the Adipo-Chondro-Osteo differentiation kit (Human mesenchymal stem cell functional identification kit, R&D Systems, Minneapolis, USA). Following the differentiation period, adipogenic, osteogenic, and chondrogenic cells were examined using Oil Red, Alizarin Red S, and Alcian Blue, respectively. The wells were analyzed using an inverted microscope (TCM 400), and imaging was performed using a digital camera (Canon, Woodhatch, UK) [22].

#### GMSCs surface markers expression

Flow cytometry analysis detected the surface antigens CD34, CD45, CD73, CD105, and HLA-DR using a Facial Action Coding System (FACS) Caliber Flow Cytometer (Cytofex, Beckman Coulter) [23].

### MTT assay

Cell viability was measured using the MTT assay. The test was conducted using the guidelines outlined in ISO 10993-5 [24]. The GMSCs were placed in 96-well plates at 1 × 10^5^ cells/ml and cultured for 24 h. The culture medium was extracted from the wells, and 100% extractions from samples or serial dilutions of extractions using culture medium (50%, 25%) in a volume of 100 µL were added to each well. After 24 and 72 h, the culture medium was extracted and substituted with 50 µL of MTT solution in phosphate-buffered saline (PBS, 1 mg/mL). The MTT solution was removed afterwards, and 100 µL of isopropanol was introduced into each well. The plates were agitated until complete dissolution of all crystals occurred. The absorbance was quantified using a spectrophotometer (Epoch, BioTek, Winooski, VT, USA) set to measure light intensity at a wavelength of 570 nm. Six wells were used per extract group, and each test was conducted in triplicate. The absorbance values obtained from each well were normalized to the control group (cells cultured in medium only), and cell viability was calculated as a percentage using the formula: cell viability (%) = (absorbance of test/absorbance of control) × 100.

### Scanning electron microscopy (SEM)

The cells were placed in 6-well plates on the composite discs and cultivated for 72 h. Afterward, a 4% glutaraldehyde solution was employed to fix the cells for 2 h at a temperature of 4 °C. For critical point drying, the samples were dehydrated by sequentially exposing them to increasing concentrations of ethanol: 25, 50, 75, 95%, and finally 100%, with each concentration being applied for 5 min. The samples underwent sputter-coating with gold using a current of 15 mA for 4 min. Scanning electron microscopy (SEM, Leo Supra 55) was used to study cell-free discs and discs with grown cells.

### q-PCR

The expression of inflammatory marker genes was quantified using RT-qPCR analysis after 7 days of incubation. IL-1β (Interleukin-1 beta) and TGF-β (Transforming Growth Factor beta) were the primary genes analyzed. The analysis was performed on a sample size of three (*n* = 3). The approach involved extracting total mRNA from each sample using the QIAGEN RNA Extraction kit (QIAGEN GmbH, Hilden, Germany) following the manufacturer’s instructions. The cDNA obtained was amplified and quantified using SYBR Green Supermix (Bio-Rad) on an RT-qPCR system manufactured by Bio-Rad. The mRNA expression levels were standardized relative to β-actin [25]. The primer sequences for the genes are provided in Table 2. The measurements of all samples were conducted three times.

**Table 2:** Primer sequence for the genes used in the study

Primer	Forward sequence	Backward sequence
IL-1β	AGCTGGAGAGTGTAGATCCCAA	TGTTTTCTGCTTGAGAGGTGCT
TGF-β	GGATACCAACTATTGCTTCAGCT	AGGCTCCAAATGTAGGGGCAGGG

### Statistical analysis

Data were statistically analyzed using one-way ANOVA followed by Tukey’s post-hoc tests to identify significant differences between groups. Multifactorial analysis of variance (ANOVA) was performed to examine the effects of polishing protocol (one-step, two-step, multi-step), paste application (with vs. without polishing paste), and surface roughness (Ra) on the viability of GMSCs. Statistical significance was set at *p* < 0.05.

## Results

### Surface roughness

**Fig. 2:**
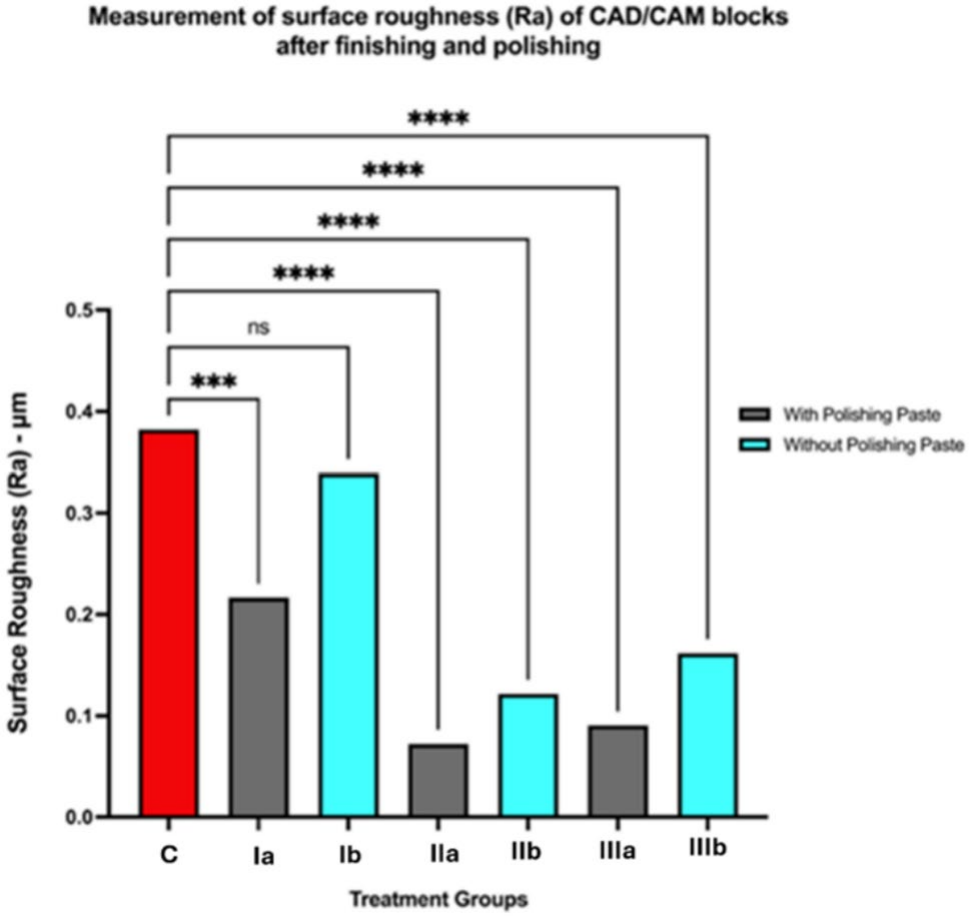
Bar chart showing surface roughness (Ra) values of different finishing and polishing groups

We compared the mean values of surface roughness (Ra) of all different finishing and polishing systems to the baseline value of the control group to confirm whether any of them had any differences in the surface roughness of the CAD/CAM blocks. There was a significant difference between all groups against the control with a *p*-value (*p* < 0.001) except for the one-step group without polishing paste, where it showed a non-significant difference with a *p*-value (*p* = 0.511) and a mean difference from the control of 0.03. All groups had low Ra values, with the highest value (0.37 μm) in the control, followed by one step without polishing paste with a mean value of 0.33 μm, followed by one step with polishing paste with a mean value of 0.22 μm. Two and multi-step without polishing paste had a mean value of 0.12 and 0.16 μm, respectively, then decreased to a lower value in the multi-step with polishing paste group, with a mean value of 0.09 μm. The lowest value was in the two-step group with a mean value of 0.07 μm (Table 3 and Fig. 2).

**Table 3:** Descriptive statistics for the surface roughness values against the control. PP = polishing paste

Groups	Number of values	Minimum	Maximum	Range	Ra (μm) mean	Std. error of mean
Control	8	0.29	0.42	0.12	0.37 ± 0.04	0.01
One-step with PP	8	0.19	0.27	0.08	0.22 ± 0.03	0.01
One-step without PP	8	0.22	0.41	0.19	0.33 ± 0.06	0.02
Two-step with PP	8	0.06	0.09	0.03	0.07 ± 0.01	0.003
Two-step without PP	8	0.12	0.14	0.02	0.12 ± 0.01	0.002
Multi-step with PP	8	0.08	0.10	0.02	0.09 ± 0.01	0.003
Multi-step without PP	8	0.13	0.18	0.05	0.16 ± 0.02	0.006

### GMSCs isolation and characterization

**Fig. 3:**
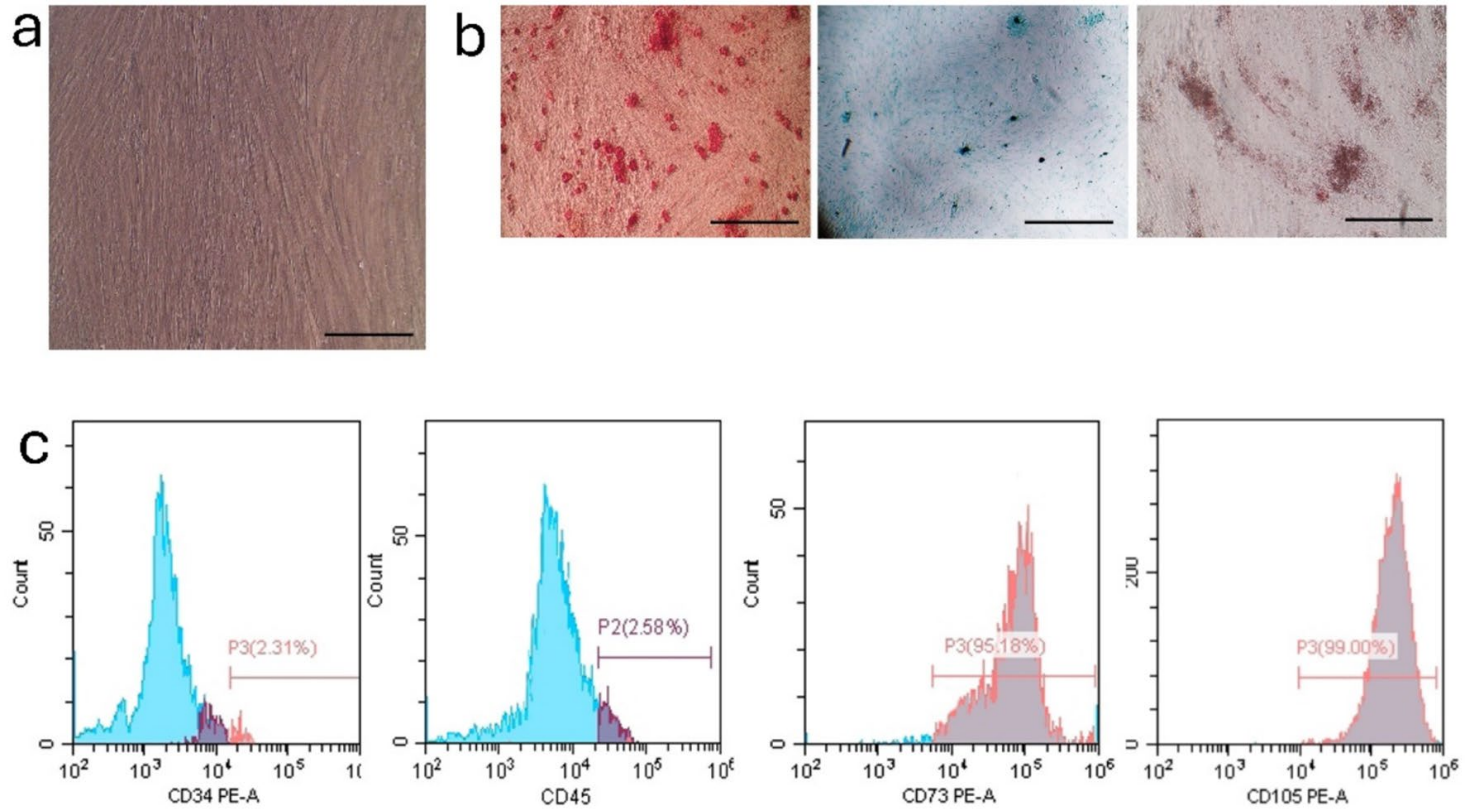
Characterization of GMSCs. **a** Morphological appearance. **b** Multilineage differentiation potential into osteogenic, chondrogenic, and adipogenic lineages. **c** Flow cytometry data of surface antigens CD34, CD45, CD73, and CD105

Under a light microscope, the cells harvested from cultured gingival biopsies were shown to be spindle-shaped (Fig. 3a). Their ability to undergo osteogenesis, chondrogenesis, and adipogenesis corroborated their pluripotency (Fig. 3b).

Flow cytometry data revealed that GMSCs exhibited a positive expression of mesenchymal surface molecular markers CD73 and CD105, whereas they showed a weak expression of hematopoietic surface markers CD34 and CD45 (Fig. 3c).

### MTT assay

**Fig. 4:**
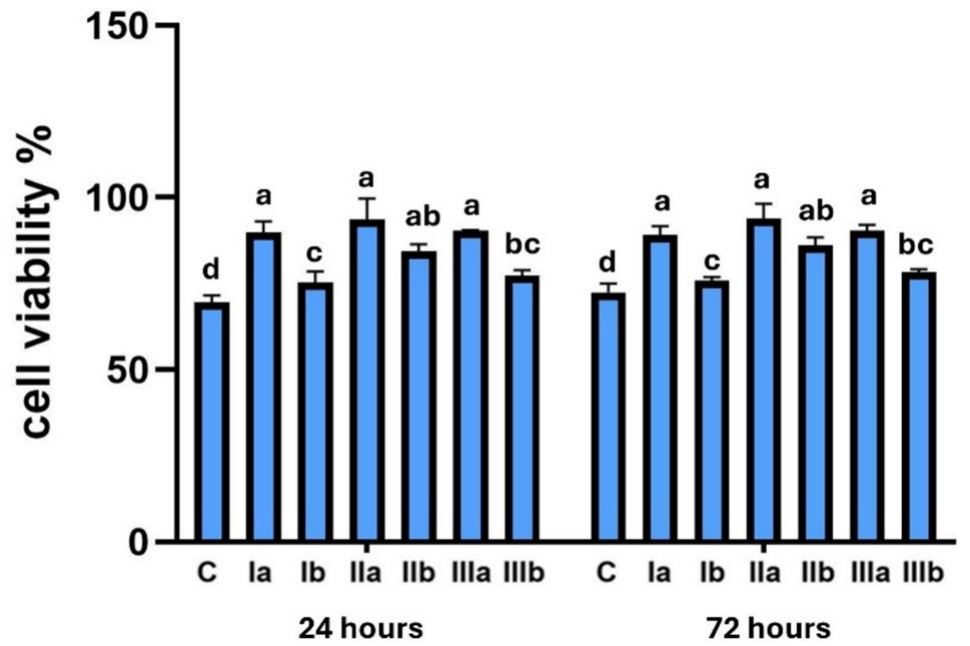
The graph represents the mean and standard deviation results of the MTT assay measuring cell viability as a percentage across different experimental groups of undiluted extracts after 24 and 72 h. Different letters indicate statistically significant differences (*p* < 0.05)

The results of the undiluted extracts at 24 and 72 h demonstrated that polished composites with polishing paste (Groups Ia, IIa, IIIa) exhibited significantly higher cell viability than their unpolished counterparts, with Group IIa having the highest viability overall. In contrast, polished composites without polishing paste (Groups Ib, IIb, IIIb) displayed reduced cell viability, with Group Ib recording the lowest among them. Notably, the control group exhibited the least cell viability, which was statistically lower than all experimental groups (Fig. 4).

Multifactorial analysis of variance (ANOVA) revealed a statistically significant main effect of polishing protocol on cell viability (*p* < 0.001) and a main effect of paste application (*p* = 0.027). Additionally, surface roughness (Ra) was a significant covariate (*p* = 0.013), indicating that smoother surfaces were associated with higher cell viability. A significant interaction between polishing protocol and paste application was also observed (*p* = 0.012), suggesting that the effect of paste differed depending on the protocol used.

### Scanning electron microscopy (SEM)

SEM examination after 7 days revealed that the control group exhibited a composite surface without any observed stem cell attachment, while the polished composites with polishing paste (Groups Ia, IIa, IIIa) showed better cell attachment and spread morphology due to the smoother surfaces provided by polishing. SEM images revealed a dense layer of well-adhered cells with extensive filopodia and lamellipodia, indicating strong attachment.

**Fig. 5:**
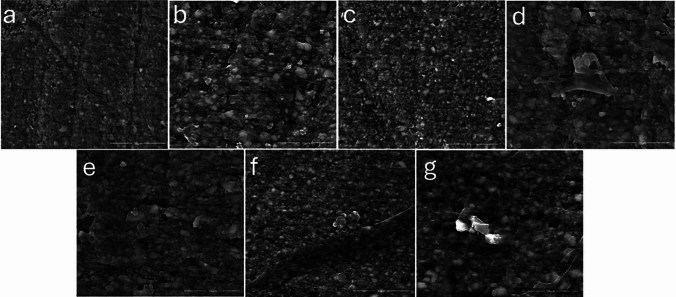
SEM analysis of GMSCs attachment to composite discs of the control group c (**a**), group Ia (**b**), group Ib (**c**), group IIa (**d**), group IIb (**e**), group IIIa (**f**), and group IIIb (**g**)

Polished composites without polishing paste (Groups Ib, IIb, IIIb) showed poorer cell attachment, with fewer cells exhibiting spread morphology. The rougher surfaces lead to fewer attachment points and less optimal cell adhesion. SEM images revealed cells with a more rounded morphology, indicating weaker attachment (Fig. 5).

### PCR

**Fig. 6:**
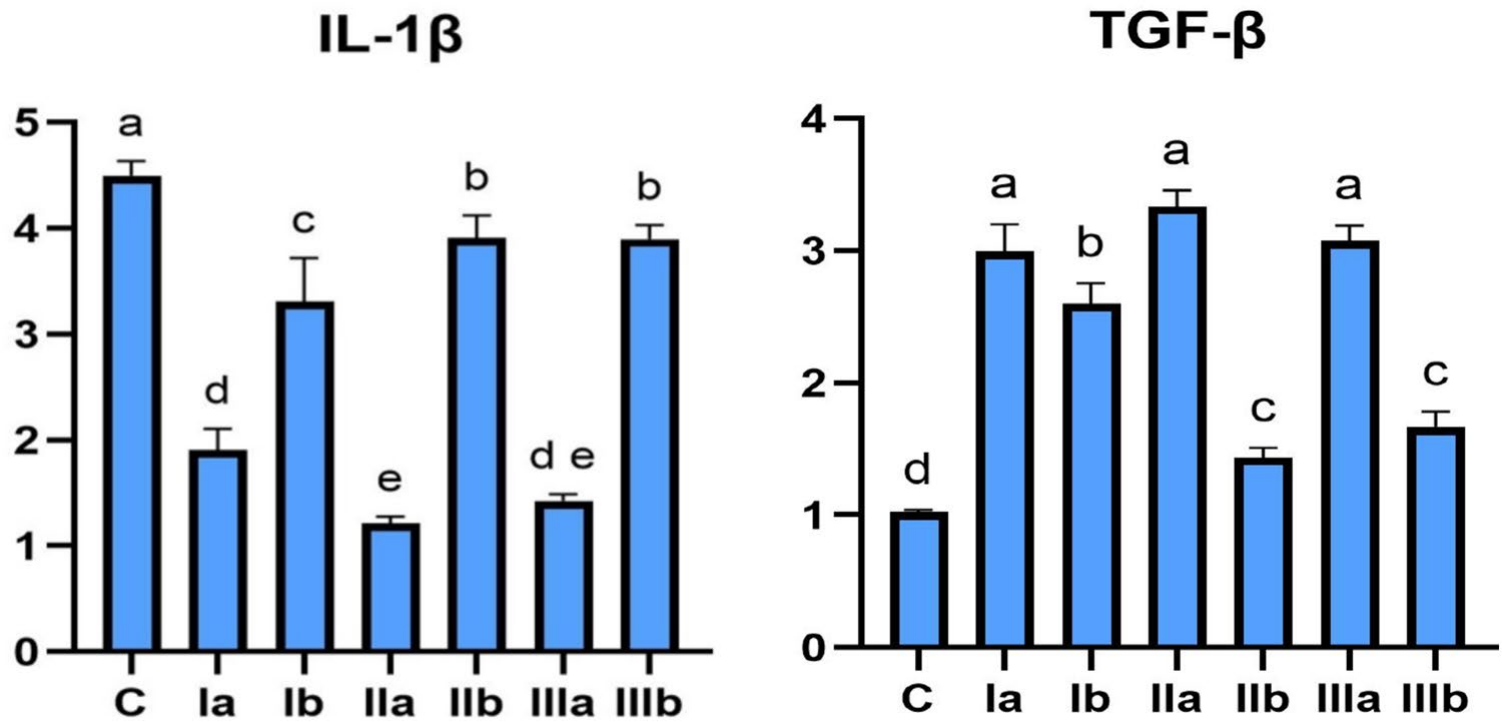
The graph displays the fold change in expression levels of IL-1β and TGF-β in gingival mesenchymal stem cells for different experimental groups. Different letters indicate statistically significant differences (*p* < 0.05)

q-PCR analysis of inflammatory gene expression revealed distinct differences among groups. IL-1β levels were highest in polished composites without polishing paste (Groups Ib, IIb, IIIb), indicating a heightened inflammatory response. In contrast, polished composites with polishing paste (Groups Ia, IIa, IIIa) demonstrated notably lower IL-1β expression, suggesting reduced inflammation. The control group showed the highest level of IL-1β, which was significantly higher compared to other groups (*p* < 0.05). Regarding TGF-β, both one-step groups (Ia and Ib) exhibited the highest expression levels, reflecting superior biocompatibility, while multi-step groups (II and III) showed variable expression. The control group recorded the lowest TGF-β levels, exhibiting a statistically significant difference compared to all other experimental groups (*p* < 0.05) (Fig. 6).

## Discussion

CAD/CAM blocks are increasingly used to fabricate dental restorations due to their numerous advantages, including high precision, accuracy, and durability. Indirect composite CAD/CAM blocks are prefabricated blocks of composite resin that are milled into dental restorations using a CAD/CAM machine. They offer several advantages over direct composite restorations, including superior mechanical properties, better marginal adaptation, and increased durability as they are less likely to chip or fracture. Furthermore, the physical, optical, and mechanical properties of the indirect CAD/CAM blocks have been enhanced. Compared to the composite CAD/CAM blocks, the ceramic is susceptible to degradation, indicating a low resistance to the staining effect and possible adverse biological effects [26]. Degradation of composite resins is associated with water absorption, chemical reaction, diet, poor oral hygiene, and surface roughness of the restoration [27]. The texture of the polished surface is affected by many factors, such as the type of resin composite, the finishing and polishing tool, the polishing paste used, and the procedure parameters. During the process of finishing and polishing, which are the final steps in the fabrication of the restoration to improve its surface characteristics and appearance, the optical properties and color stability were indeed affected by surface changes that occurred during the finishing and polishing process. Accordingly, these steps determine the surface smoothness and its susceptibility to react and attach to the surrounding gingival tissues [28].

Biocompatibility, particularly the ability of a material to support appropriate cellular attachment and proliferation without eliciting a detrimental response, is paramount. The surface characteristics of dental composites, influenced by their chemical composition and the finishing/polishing procedures used, play a crucial role in this context. A smooth, well-polished surface may favor better cell attachment and proliferation, potentially leading to improved tissue integration and healing. There is a positive correlation between Gingival stem cell attachment and viability, with both factors inversely related to inflammatory gene expression (specifically IL-1β). Good attachment and high viability are associated with lower IL-1β (reduced inflammation) and higher TGF-β (enhanced healing). These relationships underscore the importance of optimizing dental composite surface properties to promote biocompatibility and favorable cellular responses.

Concerning resin composite, various finishing and polishing systems are available that can be categorized according to their materials, abrasive size, desired smoothness, cost, and sequential steps to be followed. Following the manufacturer’s instructions is essential once the proper finishing and polishing system has been chosen. A smooth surface finish is critical to the long-term clinical success of restorative materials. Various finishing and polishing systems, such as silicone wheels, pastes, and aluminum oxide discs, have been developed to achieve this [14]. The type of finishing and polishing system that is best for a particular CAD/CAM block depends on the material of the block, the desired surface finish, the pressure applied, and the presence or absence of polishing paste as a lubricant [13]. One-step systems are the simplest and fastest to use. Two-step systems include a smoothing pre-polishing and polishing step, while multistep systems have an additional step, an extra smoothing step. Different systems with more than one step require sequential use of the tools to obtain the optimum results.

The type of composite that was used in this study was composite CAD/CAM blocks because this material is very promising, with many advantages, such as high homogeneity and degree of conversion, with low water sorption, solubility, and wear resistance compared to direct composite resins [29]. Moreover, they have a greater resistance to degradation and staining than direct resins [8]. These factors resulted in better stability and mechanical properties [1]. All these properties are due to their fabrication process, which was done under high temperatures and pressure. According to a study by Alamoush et al. in 2022, the resin composite blocks Cerasmart and Brilliant Crios exhibited lower sorption values than Lava Ultimate. The usage of Brilliant Crios blocks showed high performance when compared to other composite blocks [1]. In addition, a single brand of CAD CAM composite blocks was selected in this study to minimize the tested variables and to emphasize the effect of different polishing systems and protocols [30, 31].

Surface roughness of restorative materials is commonly evaluated using scanning electron microscopy (SEM) and various optical or mechanical profilometers. For many years, mechanical profilometers have been the favorite method of measuring surface roughness since they donot require any preparation time and allow for many measurements at different periods [18]. However, Ra is the standard and most commonly used parameter for measuring and reporting dental composite surface roughness in the literature, due to its widespread usage, clinical correlation, and feasibility of measurement. There is no significant argument to use other parameters in isolation without Ra. However, complementary parameters may be used for more accurate characterization, and this could be more relevant in a study that emphasizes only the surface roughness, which is not the case in the current study. Moreover, due to its simplicity and well-established clinical relevance, the Ra parameter was used [32–34].

Regarding the effect of different finishing and polishing systems on surface roughness when compared to the control, all groups were statistically different from the control with a *p*-value < 0.0001, except for the one-step group without polishing paste, where it showed a non-significant difference with a *p*-value (*p* = 0.511) and a mean difference from the control of 0.03. This could be attributed to the fact that the control specimens were subjected to disc cutting during specimens’ preparation from the CAD/CAM blocks. In addition, pre-finishing preparation with the high-speed yellow-coded stone caused a rough surface, triggering more surface staining. The wear mechanism during finishing and polishing is mostly abrasive wear. Surface deterioration with groove and scratch formation, potentially progressing to Hertzian fractures in certain cases, characterizes the observed damage. These results agreed with Aydin et al. in 2021 [18] as they found that the control that did not receive finishing and polishing application showed the highest amount of discoloration due to increased surface roughness. The one-step system is composed of silicon tips impregnated with tiny diamond particles (10 μmm) that might affect the smoothening effect of the system on the specimen’s surface, as the system is mainly used for polishing.

Another explanation might be the low heat generation obtained during its use as a coolant, which was applied, and the low-speed range recommended by the manufacturer (17,500 rpm). Heat generation emphasizes the smoothening effect of the finishing and polishing systems by melting the outermost layer of the restoration, obtaining a smoother surface. This assumption might need sequential tools with different abrasive particle sizes descending from coarse to fine or super-fine, which is not present in this system, as it is a one-step system. Additionally, the high mechanical properties of the CAD/CAM composite blocks might be the reason for the inability of the one-step system to produce a smooth surface. Using the polishing paste with the one-step system (0.22 μm) did not improve the results for the same reasons. These findings agreed with those of Dennis et al. in 2021 [13], Paolone et al. in 2020 [35], Bansal et al. in 2019 [36] found that the one-step polishing protocol induced heavily grooved, deeply scratched surface topographies on all the tested groups. On the contrary, Kumari et al. in 2015 disagreed with these results as they stated that a one-step polishing system with diamond polishing paste produces a smoother surface compared to a multi-step system, and this was due to the difference in the material tested and used [37].

On the other hand, the sequential steps of finishing and polishing systems, whether two-step or multistep, significantly improved the roughness results. The best results were obtained with the two-step system. This could be explained by the effect of abrasive particles impregnated in the elastomeric tools. Using different sizes of abrasive particles in the finishing and polishing tools improves the smoothening competence. The medium particles (35 μm) are responsible for removing the outer rough layer resulting from the previous contouring step. Subsequently, the smoothening effect of the fine abrasive particles (15 μm) became easier and more efficient. Furthermore, the smoother surface obtained may be due to the hardness of the abrasive particle used (diamond particles). Effective finishing systems rely on abrading particles with superior hardness compared to the filler particles in the polished material. In cases where this hardness disparity is absent, the polishing tool preferentially removes the softer resin matrix, leaving protruding filler particles and a rougher surface [36]. Moreover, the design of the tool might be the reason, as the spiral wheels provide more clearance space to remove the finished debris along with the coolant application, emphasizing the finishing and polishing effect of the tool. The results of this study are consistent with those of Bansal et al. in 2019 [36], Sasany et al. in 2022 [38], Dennis et al. in 2021 [13], Korkut et al. in 2021 [15]. On the contrary, the results of the present study did not follow a study done by Afify et al. in 2023 [39], whose results showed that the two-step system (spiral wheels) possessed a rough surface, but this could be attributed to the different types of materials that were used, as they used bulk fill resin composite.

Concerning the multistep system, it obtained better results than the one-step but less than the two-step. This might be due to the sequential effect of the different abrasive grits from coarse to fine in this system. As the particle size of the coarse tools was large (75 μm), they were mainly used for contouring that requires extra pressure, causing more scratches on the surface that could not be smoothened efficiently by the subsequent two steps [medium (40 μm) then fine (20 μm)]. The medium tool particle size is still large, which might cause more surface scratches. The more scratches obtained by the coarse and medium tools, the less effective the last fine tool is in smoothing the surface. Moreover, the used system was relatively non-resilient; so, the pressure was transmitted to the polished surface that maximized the friction effect of the tool on the composite surface. Friction exerts a micro-grinding action on the material surface, leading to material removal through mechanisms such as abrasive wear, ductile deformation, and minor micro-fracturing. Additionally, the shape of the polishing tool may also contribute to these surface alterations. The disc-shaped polishing tip, with its flat and broad contact area, intensified the frictional effect on the specimen’s flat surface, thereby enhancing surface abrasion. These findings were in line with Abo-El Dahab et al. in 2022 [40], Saleh Ismail et al. in 2023 [41], and Pavlovich et al. in 2021 [42]. In contrast, the findings of the present study did not align with those of Jaramillo-Cartagena et al. 2021 [14], who reported that multistep polishing systems were the most effective. This discrepancy may be attributed to their use of the Sof-Lex disc system, which differs from the one employed in the current study. Regarding the effect of the polishing paste within all the used systems, using polishing paste showed the best results with the two-step finishing and polishing system, followed by the multistep, and finally the one-step finishing system. The same pattern can be observed as well when comparing the groups without polishing pastes only. This might be attributed to the lubrication effect as well as the abrasive content of the paste that improves the smoothing effect on the surface. As we used diamond-based polishing paste, the hardness of diamond particles improved the competence of the two-step and multistep systems. As mentioned before, the tool design of the spiral wheels allows the excess polishing paste to escape between the bristles of the tool to avoid its exaggerated effect on the surface. On the other hand, in the multi-step system, the polishing paste might overstress the surface roughness, affecting the roughness of specimens. Moreover, the one-step system was the worst system used, as mentioned before. So, using the polishing paste did not improve all the results of the system. These results were consistent with those of Aydin et al. (2021) and Yolanda et al. (2017) [43].

Regarding clinical relevance, the primary indication for indirect restorations (ceramic or composite) is in severely compromised teeth, with one of the most common conditions being subgingival cavity margins, which are directly related to the gingival tissues. Hence, we aimed to investigate the effect of the finishing and polishing step with different systems on the reaction and the inflammatory response of the gingival tissue, especially with CAD/CAM composite blocks after the milling procedure, which produces a relatively rough surface. Some operators skip the finishing and polishing step, which might affect the gingival tissue adversely.

The current findings emphasize that the finishing and polishing protocols used on CAD/CAM composite blocks significantly affect the biological behavior of GMSCs. Specifically, smoother surfaces resulting from effective polishing were associated with improved cell viability and attachment. Both MTT results and SEM observations support this. The significant effect of surface roughness (Ra) as a covariate in the multifactorial ANOVA analysis suggests that polishing indirectly influences GMSCs’ behavior by modifying the topography of the composite surface.

Smoother surfaces may enhance the adsorption of serum proteins such as fibronectin and vitronectin, facilitating integrin-mediated adhesion and promoting focal adhesion formation—key processes in stem cell attachment and survival [44, 45]. Additionally, reduced surface irregularities may lower oxidative stress and inflammatory signaling, which aligns with the observed downregulation of IL-1β expression in well-polished surfaces [46, 47].

Moreover, the high degree of polymerization in CAD/CAM blocks minimizes residual monomer release, which reduces cytotoxicity and supports higher cell viability [48]. Additionally, the stable surface chemistry of highly polymerized materials improves protein adsorption and integrin-mediated cell adhesion, facilitating better cellular responses. However, surface roughness—modulated by different polishing protocols—remains a critical factor influencing cell behavior, even among materials with similarly high polymerization.

Although surface roughness measurements and comparisons were necessary to interpret the biological effects, the primary focus of this study lies in how these surface changes influence cell viability and inflammatory responses. The differences in viability between protocols—particularly the superior outcomes with the two-step system using polishing paste—further underscore the importance of optimizing surface characteristics for favorable biological performance.

Furthermore, while quantitative analysis of GMSC adhesion was not performed, qualitative SEM evaluation provided consistent morphological evidence of improved cell spreading and attachment in smoother groups. Together, these results demonstrate that polishing is not only a mechanical or aesthetic step but a biologically impactful procedure that modulates stem cell responses. Future studies should investigate the long-term clinical relevance of these findings and quantify adhesion more precisely using standardized assays.

Additionally, the study explored the inflammatory responses elicited by different composite systems through analysis of IL-1β and TGF-β expression. IL-1β, a pro-inflammatory cytokine, was notably higher in cells exposed to unpolished composites, particularly in groups IIb and IIIb, indicating a correlation between poor surface conditions and increased inflammation. Conversely, higher TGF-β levels, indicative of favorable biological responses and tissue healing, were most pronounced in one-step polished composites (Groups Ia and Ib). This suggests that simpler composite systems with fewer procedural steps might inherently possess better biocompatibility or at least benefit more significantly from polishing procedures.

The positive correlation between cell attachment, viability, and anti-inflammatory response (higher TGF-β expression) suggests that polished composite surfaces are optimal for promoting cell health and reducing inflammation. Conversely, the inverse relationship between attachment, viability, and pro-inflammatory responses (higher IL-1β) on unpolished surfaces highlights the importance of surface finishing treatments in clinical settings.

Based on the results of this study, the null hypothesis was rejected. However, the findings must be interpreted within certain limitations. Firstly, the in vitro design and short-term analysis period restrict the direct applicability of the results to clinical scenarios. Long-term biological responses in clinical conditions, including the influence of saliva and oral biofilms on polished composite surfaces, need further investigation. Moreover, our study includes reliance on a single roughness parameter (Ra) measured by a contact profilometer, which might not fully characterize surface topography compared to optical profilometry. Additionally, the use of only one CAD/CAM composite material limits the generalizability of these results to other composite systems. Variations in polishing outcomes due to operator technique, experience, and consistency were also not evaluated. Therefore, future research should incorporate multiple composite materials and explore inter-operator variability to enhance the validity and clinical relevance of these findings.

## Conclusion

The different finishing and polishing protocols of the CAD/CAM composite blocks have a significant effect on the surface roughness of composite CAD/CAM blocks as well as the attachment, viability, and inflammatory response of gingival mesenchymal stem cells (GMSCs).

## Supplementary Information

Below is the link to the electronic supplementary material.

Supplementary file1 (DOCX 940 KB)

## Data Availability

The datasets used and/or analysed during the current study are available from the corresponding author on reasonable request.
