# Supplementary material for: Influence of different finishing and polishing protocols of composite CAD CAM blocks on surface roughness and biological response of gingival mesenchymal stem cells
Source: Odontology. 2025 Sep 13;114(3):1320–33. doi: 10.1007/s10266-025-01190-6 (PMC13319151; doi:10.1007/s10266-025-01190-6)

Supplementary figure. Mean and standard deviation results of the MTT assay measuring cell viability as a percentage across different experimental groups of diluted extracts (1:4 and 1:2) after 24 and 72 hours. Different letters indicate statistically significant differences (p<0.05).


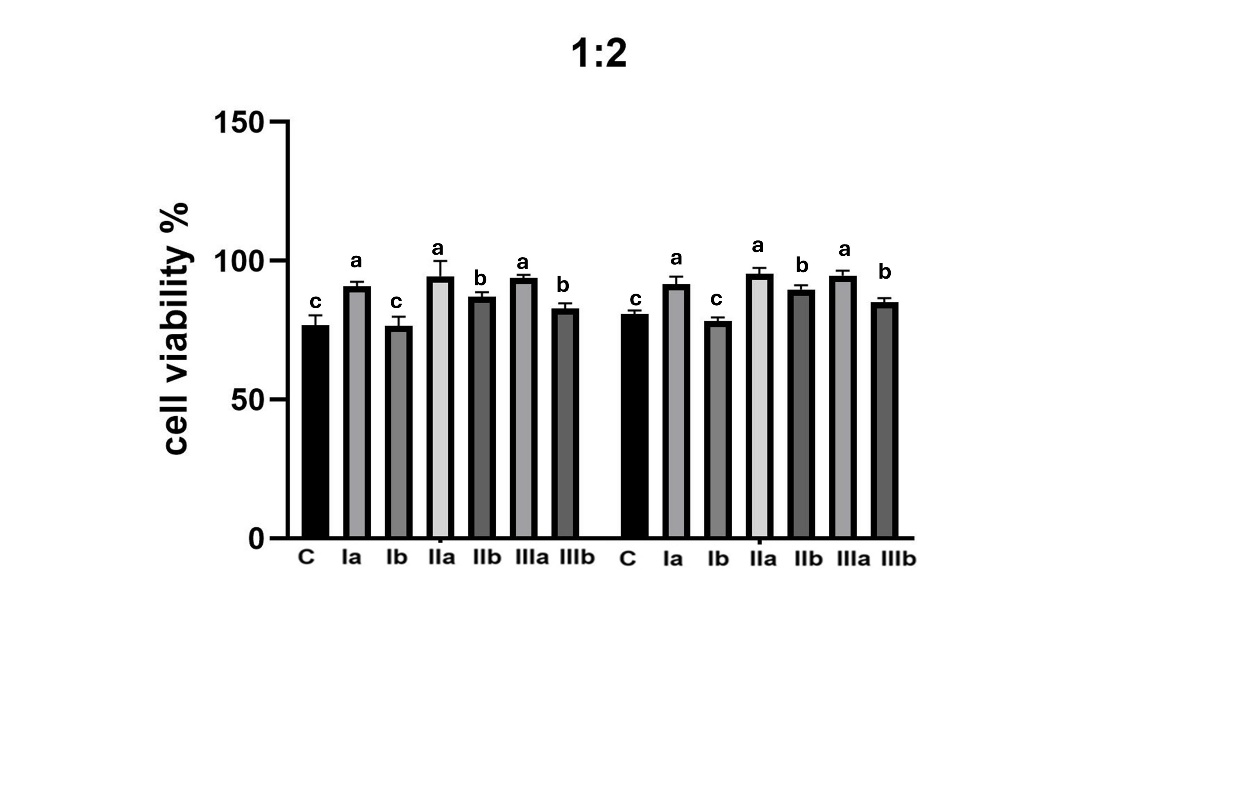

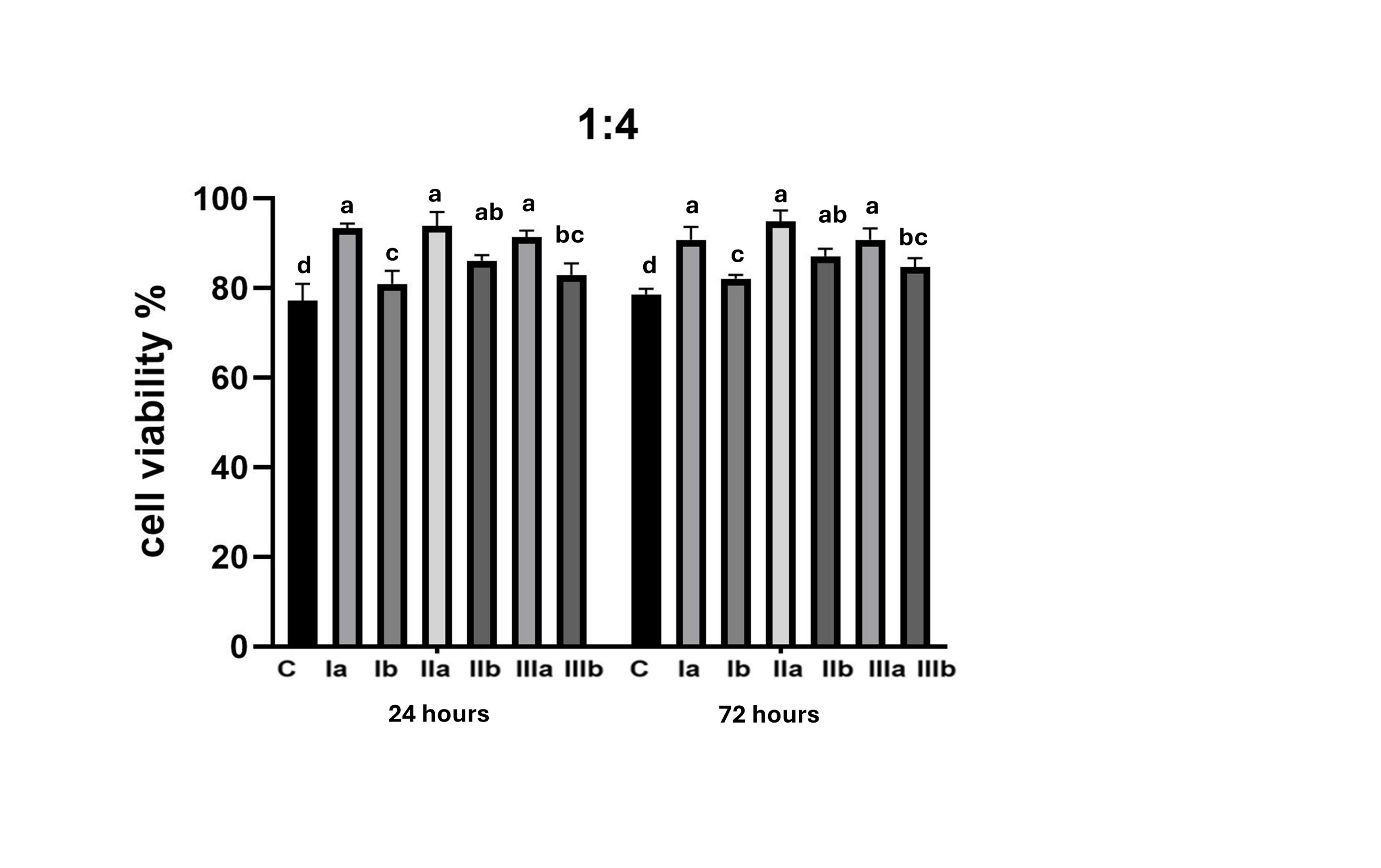

Supplement: Supplementary file 1 — Supplementary file1 (DOCX 940 KB) [file 10266_2025_1190_MOESM1_ESM.docx]
